# Subcortical neural response to click and speech stimuli in infants and young children with congenital cytomegalovirus: a preliminary study

**DOI:** 10.1590/2317-1782/e20250244en

**Published:** 2026-08-03

**Authors:** Ketilly Samilly de Souza Silva, Josefa Viviane de Moura Ferreira, Lara Louíse Pinto Câmara, Mylena Taise Azevedo Lima Bezerra, Sheila Andreoli Balen

**Affiliations:** 1 Laboratório de Inovação Tecnológica em Saúde, Universidade Federal do Rio Grande do Norte – UFRN - Natal (RN), Brasil.; 2 Departamento de Fonoaudiologia, Universidade Federal do Rio Grande do Norte – UFRN - Natal (RN), Brasil.; 3 Programa Associado de Pós-graduação em Fonoaudiologia (Mestrado) – PPgFon, Universidade Federal do Rio Grande do Norte – UFRN - Natal (RN), Brasil.; 4 Ambulatório de Pediatria, Hospital Universitário Onofre Lopes, Universidade Federal do Rio Grande do Norte – UFRN - Natal (RN), Brasil.

**Keywords:** Hearing, Electrophysiology, Cytomegalovirus, Auditory Brainstem Responses, Speech Perception, Child Development, Early Diagnosis

## Abstract

**Purpose:**

To analyze the neural response of the subcortical pathway through click and speech stimuli in infants and young children with congenital cytomegalovirus (CMVc).

**Methods:**

Cross-sectional and prospective study. The sample consisted of infants and young children with a median age of 6.43 months (Q1–Q3: 2.62–11.25) in the CMV group (G1; n=10) and 6.23 months (Q1–Q3: 1.60–12.06) in the control group (G2; n=10). All participants underwent Transient Evoked Otoacoustic Emissions (TEOAE) and bilateral peak positive pressure tympanometry. The procedures included anamnesis, Auditory Brainstem Response with click stimulus (ABR-click), and Frequency Following Response (FFR). Click ABR was performed at intensities of 80 and 30 dB nHL in both ears. FFR was recorded in the right ear with a speech stimulus /da/ of 170 ms. FFR responses were analyzed in the temporal and spectral domains, regarding neural synchrony, spectral representation, and stability of the speech response. The Mann-Whitney test or Student's t-test was applied for intergroup analysis. The Wilcoxon test was applied for intragroup analysis.

**Results:**

In the analysis of click ABR latencies and amplitudes, as well as in FFR measurements related to subcortical neural coding of speech, no statistically significant differences were observed between groups or between ears in the intergroup analysis.

**Conclusion:**

Preliminary results indicate a similar pattern of subcortical neural coding of speech and neural synchrony in infants and young children with and without CMVc.

## INTRODUCTION

Congenital infections can adversely affect infant development^(1)^. Among these infections, congenital cytomegalovirus (CMVc) is the most common congenital infection worldwide and the leading non-genetic cause of sensorineural hearing loss^(1)^.

Children who exhibit clinical signs from birth—specifically those with symptomatic congenital cytomegalovirus (CMV)—are at greater risk of developing sensorineural hearing loss, as well as neurodevelopmental delays. It is estimated that approximately 50% of symptomatic cases develop sensorineural hearing loss, while in asymptomatic cases, the prevalence is about 7%^(1)^.

Although the effects of CMVc on hearing are well recognized, gaps remain in our understanding of the neurophysiological mechanisms underlying hearing changes and their impact on typical linguistic, cognitive, and social development. Given that the early identification and treatment of central hearing loss are critical for the proper development of speech and language^(1)^, and that early diagnosis of hearing loss is directly linked to better outcomes in these areas^(2,3)^, it is essential to adopt sensitive protocols capable of detecting hearing impairments even in the early stages of development.

In this context, the *Frequency-Following Response* (FFR)—an electrophysiological measure that assesses subcortical neural encoding of complex stimuli, particularly speech—has been proposed as a promising tool for the early monitoring of auditory processing and speech disorders in at-risk populations, including infants and young children^(3)^.

In FFR, consonant-vowel (CV) stimuli, such as the syllable /da/, are the most frequently used, as they allow for a detailed assessment of the temporal and spectral characteristics of the speech signal^(4)^. Thus, the test provides information on the quality of subcortical auditory processing, based on the accuracy of neural encoding of acoustic stimuli^(5,6)^.

The study was based on the hypothesis that infants and children with CMVc exhibit alterations in subcortical neural coding of speech, as evidenced by atypical FFR responses compared with those in the control group.

Given this scenario, the present study aimed to analyze subcortical neural responses to click and speech stimuli in infants and young children with congenital cytomegalovirus (cCMV) using click ABR and FFR, with the goal of contributing to the early identification of hearing abnormalities in this at-risk population.

## METHOD

This was an observational, cross-sectional, prospective study , approved by the Institutional Review Board (CAAE: 55751222.3.0000.5292; parecer nº 5.323.957). The target population consisted of infants and young children born in public maternity hospitals. Data collection was conducted at a leading federal public health institution. All legal guardians signed the Informed Consent Form (ICF).

The total sample consisted of 20 infants and young children, divided into two groups: G1, comprising 10 participants with a confirmed diagnosis of asymptomatic congenital cytomegalovirus, identified in the first days of life by polymerase chain reaction (PCR) testing of a urine sample. Those who did not exhibit any systemic or neurological clinical signs at birth were considered asymptomatic. Group 2 consisted of 10 infants and young children with no pre-, peri-, or postnatal risk factors for hearing loss, including the absence of congenital cytomegalovirus (CMV), confirmed by a negative PCR test in the first few days of life.

The groups were matched for chronological and gestational ages to minimize biases related to auditory system maturation. The median age of the participants was 6.43 months (Q1–Q3: 2.62–11.25) in the CMVc group (G1) and 6.23 months (Q1–Q3: 1.60–12.06) in the control group (G2). All participants had a gestational age of≥ 37 weeks, ensuring that only full-term infants and young children were included.

A structured teleconsultation was conducted to gather medical history, including maternal history, delivery conditions, neuropsychomotor development, and the infants’ pathophysiological history. These data informed the composition of the control group and optimized the duration of the in-person evaluation.

The inclusion criteria for both groups were: presence of Transient-Evoked Otoacoustic Emissions (TEOEs) with a signal-to-noise ratio ≥ 6 dB at 1000, 1400, 2000, 2800, and 4000 Hz; reproducibility ≥ 90%; and tympanometry within normal limits.

For tympanometry, a 1000 Hz probe was used in infants up to 6 months of age, and a 226 Hz probe in infants and young children older than that; curves with a positive peak (type A) were considered normal. The procedures were performed using standard clinical equipment.

Infants and young children who did not meet the established audiological criteria or who exhibited any other risk factors for hearing loss (RHL) were excluded from the study^(7)^.

Auditory Brainstem Response (ABR) testing using click stimuli was initially performed at an intensity of 80 dB nHL to assess the neurophysiological integrity of the auditory pathway by analyzing the latencies and amplitudes of waves I, III, and V, as well as the interpeak intervals I–III, III–V, and I–V. The test was then conducted at an intensity of 30 dB nHL to assess the integrity of the auditory pathway up to the brainstem by detecting the V wave.

The ABR stimulation and recording parameters followed standardized clinical protocols, including artifact control and an appropriate analysis window for identifying responses.

The Frequency Following Response (FFR) was recorded using the synthetic speech stimulus /da/ presented at 80 dB nHL, delivered exclusively to the right ear via in-ear headphones. The stimulus had a total duration of 170 ms, including the onset, consonant-vowel transition, and sustained vowel portions, and was recorded in multiple averages, totaling 4,000 sweeps.

The acoustic parameters of the FFR stimulus and acquisition were performed following protocols widely described in the literature, enabling analysis of subcortical neural encoding of speech in the time and frequency domains.

During the recordings, artifact levels were kept below 10%, and data were collected during natural sleep. The electrodes were positioned with the active electrode at Cz, the reference electrode at the right mastoid (M1), and the ground electrode at the left mastoid (M2).

The time- and frequency-domain analyses of the FFR were performed using a MATLAB script previously validated by the research group led by Prof. Carles Escera (University of Barcelona, Spain).

The normality of the data was assessed using the Shapiro-Wilk test. Parametric or nonparametric statistical tests were performed according to the data distribution. Student’s t-test was used to compare Click ABR scores between groups. Mann-Whitney and Wilcoxon tests were used to analyze FFR scores between and within groups. The age variable was compared between the groups using the Mann-Whitney test, and no statistically significant difference was observed (p=0.79). A significance level of p < 0.05 was adopted.

## RESULTS

This study analyzed the click ABR and FFR responses of 20 infants and young children, including 13 females (65%) and 7 males (35%). A comparative analysis of age between the groups was performed. No statistically significant differences were observed between the groups (p = 0.79); the median age was 6.43 months (Q1–Q3: 2.62–11.25) in Group 1 and 6.23 months (Q1–Q3: 1.60–12.06) in Group 2.

In all infants and young children, waves I, III, and V were observed in the click ABR recorded at 80 dB nHL, and wave V was observed at 30 dB nHL.

Table 1 presents an analysis of the absolute wave latencies and interpeak intervals, analyzed by ear in both groups. The inferential analysis did not reveal any statistically significant differences between the groups for any of the measures analyzed, except that the mean latency of right-ear wave I in Group 1 was lower than that in Group 2, with a large effect size (Cohen’s d = 1.43), suggesting little overlap between the groups’ distributions.

**Table 1:** Descriptive and inferential analysis of the absolute latency of waves I, III, and V, as well as their amplitudes and the I-III, III-V, and I-V peak-to-peak intervals at an intensity of 80 dB nHL per ear for both groups

	**G1 (n = 10)**				**G2 (n= 10)**				p-value (G1 RE X G2RE)*	p-value (G1 LE X G2LE)*
	**RE**		**LE**		**RE**		**LE**			
	**Average**	**sd**	**Average**	**sd**	**Average**	**sd**	**Average**	**sd**		
**Waves I**	1.49	0.0994	1.54	0.137	1.60	0.0685	1.63	0.0825	0.007^*^,^**^	0.110
**Waves III**	4.29	0.412	4.36	0.507	4.23	0.307	4.31	0.422	0.739	0.813
**Waves V**	6.41	0.539	6.43	0.634	6.19	0.420	6.29	0.524	0.322	0.610
**Interpeak I-III**	2.79	0.376	2.72	0.470	2.63	0.268	2.69	0.390	0.259	0.858
**Interpeak III-V**	2.13	0.235	2.07	0.247	1.96	0.198	1.99	0.206	0.107	0.414
**Interpeak I-V**	4.92	0.479	4.89	0.586	4.58	0.388	4.67	0.499	0.103	0.378
**Ampl-I**	0.348	0.214	0.369	0.256	0.335	0.144	0.346	0.140	0.933	0.806
**Ampl-III**	0.304	0.185	0.310	0.199	0.360	0.152	0.390	0.153	0.469	0.326
**Ampl-V**	0.461	0.235	0.452	0.171	0.554	0.178	0.523	0.212	0.331	0.421

* Student's t-test for independent data;

** Cohen's d = 1.43. Wave latencies vary physiologically with age; therefore, the results should be interpreted with caution. This heterogeneity represents a recognized methodological bias in the study

**Caption:** G1 = group CMV; G2 = Control group; RE = Right ear; LE= Left ear; sd= standard deviation; Ampl = amplitude

When comparing latency, amplitude, and inter-peak intervals between ears within groups, no significant differences were found in the Wilcoxon test.

Table 2 presents the results of the Frequency Following Response (FFR) using a 170-ms /da/ speech stimulus, including analyses in the time and frequency domains for both groups.

**Table 2:** Descriptive and inferential analysis of the parameters in the time and frequency domains for the sample studied by group

**Analysis parameters FFR - /da/ 170 ms**	**G1**			**G2**			**p-value**
	**n=10**			**n=10**			
	**Q1**	**Med**	**Q3**	**Q1**	**Med**	**Q3**	
**Pre-stimulation (RMS) (μv)**	0.02	0.03	0.05	0.03	0.04	0.06	0.315
**Neural lag (ms)**	9.62	10.1	11.2	9.04	10.1	11.4	0.850
**Spectral amplitude (μv)**							
**Consonant transition (10 to 57 ms)**							
**F0**	0.01	0.02	0.04	0.02	0.03	0.04	0.247
**HH5-6**	0.002	0.004	0.005	0.004	0.004	0.007	0.579
**Vowel (57 to 170 ms)**							
**F0**	0.01	0.004	0.005	0.0238	0.0327	0.0468	0.481
**HH6**	0.002	0.003	0.005	0.004	0.005	0.008	0.218

**Caption:** G1 = group CMV; G2 = Control group; Q1= percentile 25; Med = median; Q3 = percentile 75

Spectral measurements of FFR, both in the consonantal and vowel portions of the stimulus, showed that the amplitude values of the fundamental frequency (F0) and its harmonics were similarly distributed across the groups. No statistically significant differences were found between the groups for any of the measures analyzed.

## DISCUSSION

The results of this study indicated a similar pattern of neural responses in the subcortical auditory pathway in infants and young children with congenital cytomegalovirus (cCMV) compared with the control group, as measured by both click ABR and Frequency Following Response (FFR). These findings suggest that the functional integrity of the subcortical auditory pathway was preserved at the time of assessment under the conditions studied.

ABR analysis revealed the presence of waves I, III, and V, as well as the I–III, III–V, and I–V peak-to-peak intervals in both groups, with values consistent with those described in the literature for similar protocols^(8,9)^, indicating intact neural conduction to the brainstem. The slight variation observed across studies may be attributed to visual analysis and manual marking of the waves, the equipment used, and the maturation process of the auditory pathway. Since the present study covers a wide age range (from 20 days to 3 years and 7 months), it is known that latencies and interpeak intervals decrease with increasing age ^(9)^.

Furthermore, the presence of the V wave at an intensity of 30 dB nHL suggests auditory responses within the expected range of overall hearing sensitivity. However, it should be noted that click ABR does not allow for the estimation of specific tonal thresholds by frequency.

It is important to note that the groups had similar median ages at the time of assessment, allowing control for age in the intergroup comparison. Given that subcortical neural responses to click and speech stimuli are influenced by maturation during the first two years of life ^(9)^, age-matching minimized biases related to auditory pathway maturation, ensuring that the observed differences were more directly related to the clinical condition under investigation.

The only significant finding from the statistical analysis was that the mean latency of the right ear’s wave I was significantly shorter in Group 1 than in Group 2, suggesting a faster neural response in infants and young children with CMVc. This preliminary finding should be considered in the context of the ongoing cross-sectional study and the longitudinal follow-up of these participants. Although the identification of click ABR waves was performed independently by two experienced raters, it is not possible to completely rule out the influence of visual judgment on the manual scoring of responses, especially given the small difference between the group means (G1: 1.49 ms, SD = 0.09; G2: 1.60 ms, SD = 0.06), which may limit the sensitivity for detecting subtle differences. On the other hand, the large effect size (Cohen’s d = 1.43) suggests that this finding warrants consideration. However, it should be interpreted with caution, given the number of comparisons and the lack of multiple-testing correction.

In the analysis of FFR neural lag, the observed values ranged from 9.62 to 10.1 ms in Group 1 and from 9.11 to 10.2 ms in Group 2, with no statistically significant differences found. Given that latencies ranging from 3 to 10 ms are described as typical in infants^(10)^, both groups exhibited latencies consistent with an appropriate pattern of subcortical neural synchrony, and no delays in the neural response to speech stimuli were observed in this sample.

The spectral width of the f0 of the vowel and the consonant produced spectrally more robust responses compared to the high harmonics, except for G1 in the vowel, but showed no significant difference between the groups. The stronger response may be explained by the fact that, starting in utero, infants are exposed to low-frequency sounds, with high frequencies filtered out^(3)^. This early exposure allows them to identify robust f0s at birth^(3)^.

The fact that G1 exhibited numerically lower values in the spectral amplitude of the vowel’s f0 may suggest a distinct modulation pattern in the encoding of f0 in the sustained portion resulting from CMV. However, a larger sample size is needed to confirm this finding.

In this study, infants and young children with other risk factors for hearing loss (RHL)^(7)^, as well as prematurity, in addition to CMVc, were excluded. This methodological strategy made it possible to isolate the effect of CMV on auditory responses, thereby aiding in the interpretation of the findings. However, the lack of differences between the groups should be interpreted with caution, as strict control of the variables may have reduced the variability observed in real-world clinical settings.

Although no studies have been identified that jointly evaluate click ABR and FFR in infants with CMVc, research involving other congenital infections^(11,12)^ has reported results similar to those of the present study. These findings may be related both to the methodological approaches used and to the timing of the assessment, since CMVc is characterized by delayed and fluctuating onset of hearing loss^(2,13,14)^.

In addition to hearing changes, CMVc may be associated with neurological impairments and congenital malformations^(1)^. Cohort studies have shown an increased risk of delays in language development among children exposed to CMV in utero^(13)^. In this context, FFR—by reflecting the neural coding of the fundamental frequency (F0) and the initial formants (F1)—provides relevant information about the neural mechanisms underlying phonemic discrimination and may contribute to monitoring language development in at-risk populations^(15)^.

In the sample studied, the initial hypothesis that infants and young children with CMVc would exhibit atypical neural responses on ABR and FFR compared to the control group was not confirmed. However, several limitations should be taken into account, including the small sample size and the cross-sectional design, which may have limited the detection of subtle differences, especially given the late-onset nature of hearing loss associated with CMVc. Furthermore, using the click ABR test only at 80 and 30 dB nHL made it impossible to estimate frequency-specific hearing thresholds.

The lack of complementary procedures, such as ABR for specific frequencies, Visual Reinforcement Audiometry (VRA), neurodevelopmental assessments (e.g., Bayley III), and functional neuroimaging methods, also limits the scope of the conclusions.

In summary, although the findings indicate that subcortical auditory responses were intact at the time of evaluation, it is not possible to predict the future auditory and language prognosis for infants and young children with CMVc. Thus, this study underscores the importance of longitudinal audiological and electrophysiological monitoring of this population, given the risk of delayed hearing loss and potential subtle impacts on language development. The results presented are preliminary, and the study is ongoing, with plans to expand the sample size and conduct longitudinal follow-up of these participants.

## CONCLUSION

This preliminary study found a similar pattern of subcortical neural encoding of speech and neural synchrony in infants and young children with CMVc, compared with the control group. Neural responses were also similar between the ears in each group studied. This study underscores the role of the ABR click and the FFR as tools for monitoring children with CMVc.
