# Supplementary material for: Subcortical neural response to click and speech stimuli in infants and young children with congenital cytomegalovirus: a preliminary study
Source: Codas. 2026 Aug 3;38(5):e20250244. doi: 10.1590/2317-1782/e20250244en (PMC13445991; doi:10.1590/2317-1782/e20250244en)
Supplement: Translation

# Translation: Resposta neural subcortical aos estímulos clique e de fala em lactentes e crianças pequenas com citomegalovírus congênito: um estudo preliminar

**DOI:** 10.1590/2317-1782/e20250244pt

**Authors:** Ketilly Samilly de Souza Silva, Josefa Viviane de Moura Ferreira, Lara Louíse Pinto Câmara, Mylena Taise Azevedo Lima Bezerra, Sheila Andreoli Balen

**Affiliations:** 1 Laboratório de Inovação Tecnológica em Saúde, Universidade Federal do Rio Grande do Norte – UFRN - Natal (RN), Brasil.; 2 Departamento de Fonoaudiologia, Universidade Federal do Rio Grande do Norte – UFRN - Natal (RN), Brasil.; 3 Programa Associado de Pós-graduação em Fonoaudiologia (Mestrado) – PPgFon, Universidade Federal do Rio Grande do Norte – UFRN - Natal (RN), Brasil.; 4 Ambulatório de Pediatria, Hospital Universitário Onofre Lopes, Universidade Federal do Rio Grande do Norte – UFRN - Natal (RN), Brasil.

**Keywords:** Audição, Eletrofisiologia, Citomegalovírus, Potenciais Evocados Auditivos do Tronco Encefálico, Percepção da Fala, Desenvolvimento Infantil, Diagnóstico Precoce

## Abstract

**Objetivo:**

Analisar a resposta neural da via subcortical por meio de estímulos clique e de fala em lactentes e crianças pequenas com citomegalovírus congênito (CMVc).

**Método:**

Estudo transversal e prospectivo. A amostra foi constituída por lactentes e crianças pequenas com mediana na idade de 6,43 meses (Q1–Q3: 2,62–11,25) no grupo CMVc (G1; n=10) e de 6,23 meses (Q1–Q3: 1,60–12,06) no grupo controle (G2; n=10). Todos os participantes apresentaram Emissões Otoacústicas Evocadas por Estímulo Transiente (EOAT) e timpanometria com pico de pressão positiva bilateralmente. Os procedimentos incluíram anamnese, Potencial Evocado Auditivo de Tronco Encefálico com estímulo clique (PEATE-clique) e Frequency Following Response (FFR). O PEATE-clique foi realizado nas intensidades de 80 e 30 dB nNA, em ambas as orelhas. O FFR foi registrado na orelha direita com estímulo de fala /da/ de 170 ms. As respostas do FFR foram analisadas nos domínios temporal e espectral, quanto à sincronia neural, representação espectral e estabilidade da resposta à fala. Foi aplicado o teste de Mann-Whitney ou Teste t de Student para análise intergrupos. O teste Wilcoxon foi aplicado na análise intragrupos.

**Resultados:**

Na análise das latências e amplitudes do PEATE-clique, bem como nas medidas do FFR relacionadas à codificação neural subcortical da fala, não foram observadas diferenças estatisticamente significativas entre os grupos e entre as orelhas na análise intergrupos.

**Conclusão:**

Os resultados preliminares indicam padrão semelhante de codificação neural subcortical da fala e de sincronia neural em lactentes e crianças pequenas com e sem CMVc.

## INTRODUÇÃO

As infecções congênitas podem desencadear impactos negativos no desenvolvimento dos lactentes^(1)^. Dentre essas infecções, destaca-se o citomegalovírus congênito (CMVc), reconhecido como a infecção congênita mais frequente no mundo e como a principal causa não genética de perda auditiva sensorioneural^(1)^.

As crianças que apresentam sinais clínicos desde o nascimento, sendo estas com o CMVc sintomático, têm um maior risco para o desenvolvimento de perdas auditivas sensorioneurais, além de atrasos no neurodesenvolvimento. Estima-se que aproximadamente 50% dos casos sintomáticos evoluam com perda auditiva sensorioneural, enquanto nos casos assintomáticos essa prevalência é de cerca de 7%^(1)^.

Apesar do reconhecimento dos efeitos do CMVc sobre a audição, persistem lacunas na compreensão dos mecanismos neurofisiológicos subjacentes às alterações auditivas e de como estas impactam o desenvolvimento linguístico, cognitivo e social típico. Considerando que a identificação e o tratamento precoce do CMVc são fundamentais para o adequado desenvolvimento da fala e da linguagem^(1)^, e que o diagnóstico precoce da perda auditiva está diretamente relacionado a melhores desfechos nessas habilidades^(2,3)^, torna-se essencial a adoção de protocolos sensíveis capazes de detectar alterações auditivas ainda em fases iniciais do desenvolvimento.

Nesse contexto, o *Frequency-Following Response* (FFR), uma medida eletrofisiológica que avalia a codificação neural subcortical de estímulos complexos, especialmente de fala, tem sido proposto como uma ferramenta promissora para o monitoramento precoce do processamento auditivo e distúrbios da fala em populações de risco, incluindo lactentes e crianças pequenas^(3)^.

No FFR, os estímulos de consoante-vogal (CV), como a sílaba /da/, são os mais frequentemente utilizados, por permitirem a avaliação detalhada da representação temporal e espectral do sinal de fala^(4)^. Dessa forma, o teste fornece informações sobre a qualidade do processamento auditivo subcortical com base na fidelidade da codificação neural dos estímulos acústicos^(5,6)^.

Partiu-se da hipótese de que lactentes e crianças com CMVc apresentariam alterações na codificação neural subcortical da fala, evidenciadas por respostas atípicas no FFR, quando comparados aos do grupo controle.

Diante desse cenário, o presente estudo teve como objetivo analisar a resposta neural subcortical aos estímulos clique e de fala em lactentes e crianças pequenas com citomegalovírus congênito (CMVc), por meio do PEATE-clique e do FFR, visando contribuir para a identificação precoce de alterações auditivas nessa população de risco.

## MÉTODO

Este estudo é caracterizado como observacional, transversal e prospectivo, aprovado pelo Comitê de Ética em Pesquisa (CAAE: 55751222.3.0000.5292; parecer nº 5.323.957). A população-alvo foi composta por lactentes e crianças pequenas nascidos em maternidades públicas. A coleta de dados foi realizada em uma instituição federal de referência em saúde pública. Todos os responsáveis legais assinaram o Termo de Consentimento Livre e Esclarecido (TCLE).

A amostra total foi composta por 20 lactentes e crianças pequenas, distribuídos em dois grupos: G1, constituído por 10 participantes com diagnóstico confirmado de citomegalovírus congênito assintomático, identificados nos primeiros dias de vida por teste de Reação em Cadeia da Polimerase (PCR) em amostra de urina. Foram considerados assintomáticos os que não apresentaram sinais clínicos sistêmicos ou neurológicos ao nascimento. O G2 foi composto por 10 lactentes e crianças pequenas sem indicadores de risco pré, peri ou pós-natais para deficiência auditiva, incluindo a ausência de CMVc, confirmada por PCR negativo nos primeiros dias de vida.

Os grupos foram pareados por idade cronológica e idade gestacional, com o objetivo de minimizar vieses relacionados à maturação do sistema auditivo. A idade dos participantes apresentou mediana de 6,43 meses (Q1–Q3: 2,62–11,25) no grupo CMVc (G1) e de 6,23 meses (Q1–Q3: 1,60–12,06) no grupo controle (G2). Todos apresentaram idade gestacional ≥ 37 semanas, garantindo a inclusão apenas de lactentes e crianças pequenas nascidos a termo.

Foi realizada teleconsulta estruturada para anamnese, na qual foram coletadas informações sobre histórico materno, condições do parto, desenvolvimento neuropsicomotor e antecedentes fisiopatológicos dos lactentes. Esses dados subsidiaram a composição do grupo controle e otimizaram o tempo da avaliação presencial.

Os critérios de inclusão para ambos os grupos foram: presença de Emissões Otoacústicas Evocadas por Estímulo Transiente (EOAT) com relação sinal/ruído ≥ 6 dB nas frequências de 1000, 1400, 2000, 2800 e 4000 Hz, com reprodutibilidade ≥ 90%, e timpanometria dentro dos padrões de normalidade.

Para a timpanometria, utilizou-se sonda de 1000 Hz em lactentes até 6 meses de idade e de 226 Hz em lactentes e crianças pequenas acima dessa idade, sendo consideradas normais curvas com pico positivo ou tipo A. Os procedimentos foram realizados em equipamento clínico padrão.

Foram excluídos do estudo lactentes e crianças pequenas que não atenderam aos critérios audiológicos estabelecidos ou que apresentaram quaisquer outros indicadores de risco para deficiência auditiva (IRDA)^(7)^.

O Potencial Evocado Auditivo de Tronco Encefálico (PEATE) com estímulo clique foi realizado inicialmente na intensidade de 80 dB nNA, para avaliação da integridade neurofisiológica da via auditiva por meio da análise das latências e amplitudes das ondas I, III e V, bem como dos intervalos interpicos I–III, III–V e I–V. Posteriormente, o exame foi realizado na intensidade de 30 dB nNA, com o objetivo de verificar a integridade da via auditiva até o tronco encefálico por meio da presença da onda V.

Os parâmetros de estimulação e registro do PEATE seguiram protocolos clínicos padronizados, com controle de artefatos e janela de análise adequada para a identificação das respostas.

O *Frequency Following Response* (FFR) foi registrado utilizando o estímulo de fala sintético /da/, apresentado na intensidade de 80 dB nNA, exclusivamente na orelha direita, por meio de fones de inserção. O estímulo apresentou duração total de 170 ms, incluindo porções de onset, transição consoante-vogal e vogal sustentada, sendo registrado em múltiplas promediações, totalizando 4000 sweeps.

Os parâmetros acústicos do estímulo e de aquisição do FFR seguiram protocolos amplamente descritos na literatura, permitindo a análise da codificação neural subcortical da fala nos domínios do tempo e da frequência.

Durante as aquisições, os níveis de artefatos foram mantidos abaixo de 10%, sendo os registros obtidos em sono natural. Os eletrodos foram posicionados com eletrodo ativo em Cz, eletrodo de referência na mastóide direita (M1) e eletrodo terra na mastóide esquerda (M2).

As análises do FFR nos domínios do tempo e da frequência foram realizadas por meio de script desenvolvido em ambiente Matlab, previamente validado pelo grupo de pesquisa coordenado pelo Prof. Carles Escera (Universidade de Barcelona, Espanha).

A normalidade dos dados foi avaliada por meio do teste de Shapiro-Wilk. Foram utilizados testes estatísticos paramétricos ou não paramétricos conforme a distribuição dos dados, adotando-se o teste t de Student para comparações intergrupos das medidas do PEATE-clique e os testes de Mann-Whitney e Wilcoxon para as análises inter e intragrupo das medidas do FFR. A comparação da variável idade entre os grupos foi realizada por meio do teste de Mann-Whitney, não sendo observada diferença estatisticamente significativa (p=0,79). Adotou-se o nível de significância de p < 0,05.

## RESULTADOS

Neste estudo foram analisadas as respostas do PEATE-clique e do FFR de 20 lactentes e crianças pequenas, sendo 13 do sexo feminino (65%) e 7 do masculino (35%). Foi realizada análise comparativa da idade entre os grupos. Não foram observadas diferenças estatisticamente significativas entre os grupos (p = 0,79), sendo a mediana de idade de 6,43 meses (Q1–Q3: 2,62–11,25) no G1 e de 6,23 meses (Q1–Q3: 1,60–12,06) no G2.

Em todos os lactentes e crianças pequenas, observou-se a presença das ondas I, III e V no PEATE-clique registrado a 80 dB nNA e da onda V a 30 dB nNA.

Na Tabela 1 são apresentadas a análise das latências absolutas das ondas e dos intervalos interpicos, analisados por orelha em ambos os grupos. A análise inferencial não evidenciou diferenças estatisticamente significativas entre os grupos para nenhuma das medidas analisadas, exceto que a média da latência da onda I da orelha direita do G1 foi menor do que a do G2 com um tamanho de efeito elevado (Cohen's d = 1,43), sugerindo baixa sobreposição entre as distribuições dos grupos.

**Tabela 1:** Análise descritiva e inferencial da latência absoluta das ondas I, III e V e amplitudes, e intervalos interpicos I-III, III-V e I-V na intensidade de 80 dB nNA por orelha de ambos os grupos

	**G1 (n = 10)**				**G2 (n= 10)**				p-valor (G1 OD X G2OD)*	p-valor (G1 OE X G2OE)*
	**OD**		**OE**		**OD**		**OE**			
	**Média**	**dp**	**Média**	**dp**	**Média**	**dp**	**Média**	**dp**		
**Onda I**	1,49	0,0994	1,54	0,137	1,60	0,0685	1,63	0,0825	0,007*,**	0,110
**Onda III**	4,29	0,412	4,36	0,507	4,23	0,307	4,31	0,422	0,739	0,813
**Onda V**	6,41	0,539	6,43	0,634	6,19	0,420	6,29	0,524	0,322	0,610
**Interpico I-III**	2,79	0,376	2,72	0,470	2,63	0,268	2,69	0,390	0,259	0,858
**Interpico III-V**	2,13	0,235	2,07	0,247	1,96	0,198	1,99	0,206	0,107	0,414
**Interpico I-V**	4,92	0,479	4,89	0,586	4,58	0,388	4,67	0,499	0,103	0,378
**Ampl-I**	0,348	0,214	0,369	0,256	0,335	0,144	0,346	0,140	0,933	0,806
**Ampl-III**	0,304	0,185	0,310	0,199	0,360	0,152	0,390	0,153	0,469	0,326
**Ampl-V**	0,461	0,235	0,452	0,171	0,554	0,178	0,523	0,212	0,331	0,421

* Teste t de Student para dados independentes;

** Cohen's d = 1,43. As latências das ondas variam fisiologicamente com a idade; portanto, os resultados devem ser interpretados com cautela. Essa heterogeneidade representa um viés metodológico reconhecido do estudo

**Legenda:** G1 = grupo CMV; G2 = Grupo controle; OD = Orelha direita; OE= Orelha esquerda; dp= desvio padrão; Ampl = amplitude

Ao comparar a latência, amplitude e interpicos entre as orelhas intragrupos, não foi evidenciada nenhuma diferença significativa no teste de Wilcoxon.

Na Tabela 2 são apresentados os resultados do *Frequency Following Response* (FFR) com estímulo de fala /da/ de 170 ms, considerando as análises nos domínios do tempo e da frequência para ambos os grupos.

**Tabela 2:** Análise descritiva e inferencial dos parâmetros no domínio do tempo e da frequência na amostra estudada por grupo

**Parâmetros de análise**	**G1**			**G2**			**p-value**
**FFR - /da/ 170 ms**	**n=10**			**n=10**			
	**Q1**	**Med**	**Q3**	**Q1**	**Med**	**Q3**	
**Pré-estímulo (RMS) (μv )**	0,02	0,03	0,05	0,03	0,04	0,06	0,315
**Neural lag (ms)**	9,62	10,1	11,2	9,04	10,1	11,4	0,85
**Amplitude spectral (μv )**							
**Transição da consoante (10 a 57 ms)**							
**F0**	0,01	0,02	0,04	0,02	0,03	0,04	0,247
**HH5-6**	0,002	0,004	0,005	0,004	0,004	0,007	0,579
**Vogal (57 a 170 ms)**							
**F0**	0,01	0,004	0,005	0,0238	0,0327	0,0468	0,481
**HH6**	0,002	0,003	0,005	0,004	0,005	0,008	0,218

**Legenda:** G1 = grupo CMV; G2 = Grupo controle; Q1 = percentil 25; Med = mediana; Q3 = percentil 75

As medidas espectrais do FFR, tanto na porção consonantal quanto na porção vocálica do estímulo, os valores de amplitude da frequência fundamental (F0) e de seus harmônicos apresentaram distribuição semelhante entre os grupos. Não foram identificadas diferenças estatisticamente significativas entre os grupos para nenhuma das medidas analisadas.

## DISCUSSÃO

Os resultados do presente estudo indicaram padrão semelhante de resposta neural da via auditiva subcortical em lactentes e crianças pequenas com citomegalovírus congênito (CMVc), quando comparados ao grupo controle, tanto no PEATE-clique quanto no *Frequency Following Response* (FFR). Esses achados sugerem preservação da integridade funcional da via auditiva subcortical no momento da avaliação, nas condições investigadas.

Na análise do PEATE, observou-se a presença das ondas I, III e V e dos intervalos interpicos I–III, III–V e I–V em ambos os grupos, com valores compatíveis aos descritos na literatura para protocolos semelhantes^(8,9)^, indicando integridade da condução neural até o tronco encefálico. A leve variação observada entre os estudos pode se justificar pela análise visual e marcação manual das ondas, o equipamento utilizado, além do processo maturacional da via auditiva. Uma vez que o presente estudo tem variabilidade (20 dias a 3 anos e 7 meses), sabe-se que a diminuição das latências e dos intervalos interpicos ocorre conforme o aumento da idade^(9)^.

Ademais, a presença da onda V na intensidade de 30 dB nNA, sugere respostas auditivas dentro da faixa esperada de sensibilidade auditiva global. Entretanto, destaca-se que o PEATE-clique não permite a estimativa de limiares tonais específicos por frequência.

É importante destacar que os grupos apresentaram idades medianas semelhantes no momento da avaliação, permitindo controle da variável idade na comparação intergrupos. Considerando que as respostas neurais subcorticais aos estímulos clique e de fala sofrem influência do processo maturacional nos primeiros dois anos de vida^(9)^, o pareamento etário minimizou vieses relacionados à maturação da via auditiva, assegurando que as diferenças observadas estivessem mais diretamente relacionadas à condição clínica investigada.

O único resultado significativo constatado pela análise estatística foi que a média da latência da onda I da orelha direita foi significativamente menor no G1 quando comparado ao G2, o que aponta indícios de resposta neural mais rápida nos lactentes e crianças pequenas com CMVc. Este achado preliminar isolado deve ser observado na continuidade do estudo transversal e no acompanhamento longitudinal destes participantes. Ainda que a identificação das ondas do PEATE-clique tenha sido realizada de forma independente por dois avaliadores experientes, não é possível descartar completamente a influência do julgamento visual na marcação manual das respostas, sobretudo diante da reduzida diferença entre as médias dos grupos (G1: 1,49 ms, DP = 0,09; G2: 1,60 ms, DP = 0,06), o que pode limitar a sensibilidade para a detecção de diferenças sutis. Por outro lado, o tamanho do efeito elevado (Cohen's d = 1,43) sugere que é um achado a ser considerado. No entanto, deve ser interpretado com cautela, considerando o número de comparações realizadas e a ausência de correção para múltiplos testes.

Na análise do *neural lag* do FFR, os valores observados variaram entre 9,62 e 10,1 ms no G1 e entre 9,11 e 10,2 ms no G2, não tendo sido constatadas diferenças estatisticamente significativas. Considerando que variações entre 3 e 10 ms são descritas como típicas em lactentes^(10)^, ambos os grupos apresentaram latências compatíveis com um padrão adequado de sincronia neural subcortical, não sendo evidenciados atrasos na resposta neural ao estímulo de fala nesta amostra.

A amplitude espectral da f0 da vogal e da consoante gerou respostas espectralmente mais robustas quando comparadas aos harmônicos altos, exceto para o G1 na vogal, mas não mostrou diferença significativa entre os grupos. A maior robustez da resposta pode ser justificada pelo fato de que, desde a gestação, os lactentes serem expostos a sons de baixa frequência e ocorrer filtragem das frequências altas^(3)^. Esta exposição inicial proporciona que ao nascimento já consigam detectar f0 robustas^(3)^.

O G1 ter apresentado valores numericamente menores na amplitude espectral da f0 da vogal pode sugerir um padrão distinto de modulação na codificação da f0 na porção sustentada decorrente do CMV. No entanto, é necessário ampliar o tamanho amostral para confirmar esse achado.

Neste estudo, foram excluídos lactentes e crianças pequenas que apresentassem outros indicadores de risco para deficiência auditiva (IRDA)^(7)^, bem como prematuridade, além do CMVc. Essa estratégia metodológica permitiu isolar o efeito do CMVc sobre as respostas auditivas, contribuindo para a interpretação dos achados. No entanto, a ausência de diferenças entre os grupos deve ser interpretada com cautela, uma vez que o controle rigoroso das variáveis pode ter reduzido a variabilidade observada em contextos clínicos reais.

Embora não tenham sido identificados estudos que avaliem conjuntamente o PEATE-clique e o FFR em lactentes com CMVc, investigações envolvendo outras infecções congênitas^(11,12)^ relataram resultados semelhantes aos do presente estudo. Esses achados podem estar relacionados tanto às características metodológicas adotadas quanto ao momento da avaliação, uma vez que o CMVc se caracteriza pela possibilidade de surgimento tardio e flutuante da perda auditiva^(2,13,14)^.

Além das alterações auditivas, o CMVc pode estar associado a comprometimentos neurológicos e malformações congênitas^(1)^. Estudos de coorte demonstraram maior risco de atrasos no desenvolvimento da linguagem em crianças expostas ao CMV intraútero^(13)^. Nesse contexto, o FFR, por refletir a codificação neural da frequência fundamental (F0) e dos formantes iniciais (F1), fornece informações relevantes sobre os mecanismos neurais subjacentes à discriminação fonêmica, podendo contribuir para o monitoramento do desenvolvimento da linguagem em populações de risco^(15)^.

Na amostra estudada, a hipótese inicial de que lactentes e crianças pequenas com CMVc apresentariam respostas neurais atípicas no PEATE e FFR em comparação ao grupo controle não foi confirmada. Entretanto, algumas limitações devem ser consideradas, incluindo o reduzido tamanho amostral e o delineamento transversal, que podem ter limitado a detecção de diferenças sutis, especialmente diante do caráter tardio da perda auditiva associada ao CMVc. Ademais, a utilização do PEATE-clique apenas nas intensidades de 80 e 30 dB nNA impossibilitou a estimativa de limiares auditivos por frequência específica.

A ausência de procedimentos complementares, como o PEATE por frequência específica, a Audiometria de Reforço Visual (VRA), avaliações de neurodesenvolvimento (ex.: Bayley III) e métodos de neuroimagem funcional, também restringe a abrangência das conclusões.

Em síntese, embora os achados indiquem integridade das respostas auditivas subcorticais no momento da avaliação, não é possível inferir o prognóstico auditivo e linguístico futuro de lactentes e crianças pequenas com CMVc. Assim, o presente estudo reforça a importância do monitoramento audiológico e eletrofisiológico longitudinal dessa população, considerando o risco de perda auditiva tardia e possíveis impactos sutis no desenvolvimento da linguagem. Os resultados apresentados são preliminares, e o estudo encontra-se em andamento, com ampliação da amostra e seguimento longitudinal desses participantes.

## CONCLUSÃO

Trata-se de um estudo preliminar que indicou padrão semelhante de codificação neural subcortical da fala e de sincronia neural em lactentes e crianças pequenas com CMVc quando comparados ao grupo controle. As respostas neurais também foram semelhantes entre as orelhas em cada grupo estudado. Este estudo reforça o papel do PEATE clique e do FFR como ferramenta no monitoramento de crianças com CMVc.
